# The Pbo Cluster from *Pseudomonas syringae* pv. Phaseolicola NPS3121 Is Thermoregulated and Required for Phaseolotoxin Biosynthesis

**DOI:** 10.3390/toxins13090628

**Published:** 2021-09-07

**Authors:** Lizeth Guardado-Valdivia, Alejandra Chacón-López, Jesús Murillo, Jorge Poveda, José Luis Hernández-Flores, Luis Xoca-Orozco, Selene Aguilera

**Affiliations:** 1 Laboratorio Integral de Investigación en Alimentos, Departamento de Química y Bioquímica, Tecnológico Nacional de México, Instituto Tecnológico de Tepic, 63175 Nayarit, Mexico; yaliguardadova@ittepic.edu.mx (L.G.-V.); mchacon@tepic.tecnm.mx (A.C.-L.); 2 Institute for Multidisciplinary Research in Applied Biology (IMAB), Universidad Pública de Navarra (UPNA), Edificio de Agrobiotecnología, Avda. de Pamplona 123, 31192 Mutilva Baja, Spain; jesus.murillo@unavarra.es (J.M.); jorge.poveda@unavarra.es (J.P.); 3 Centro de Investigación y Estudios Avanzados del IPN, Departamento de Ingeniería Genética, Irapuato, 36821 Guanajuato, Mexico; jose.hernandezf@cinvestav.mx; 4 Departamento de Ingeniería Bioquímica, Instituto Tecnológico Superior de Purísima del Rincón, Purísima del Rincón, 36413 Guanajuato, Mexico; luis.xo@purisima.tecnm.mx

**Keywords:** *Pseudomonas syringae*, *Pseudomonas amygdali*, *Pseudomonas savastanoi*, phaseolotoxin, Pbo cluster, non-ribosomal peptide synthetases, genomic island, antimetabolite toxin, polyketide synthetase

## Abstract

The bean (*Phaseolus vulgaris*) pathogen *Pseudomonas syringae* pv. phaseolicola NPS3121 synthesizes phaseolotoxin in a thermoregulated way, with optimum production at 18 °C. Gene *PSPPH_4550* was previously shown to be thermoregulated and required for phaseolotoxin biosynthesis. Here, we established that *PSPPH_4550* is part of a cluster of 16 genes, the Pbo cluster, included in a genomic island with a limited distribution in *P. syringae* and unrelated to the possession of the phaseolotoxin biosynthesis cluster. We identified typical non-ribosomal peptide synthetase, and polyketide synthetase domains in several of the *pbo* deduced products. RT-PCR and the analysis of polar mutants showed that the Pbo cluster is organized in four transcriptional units, including one monocistronic and three polycistronic. Operons *pboA* and *pboO* are both essential for phaseolotoxin biosynthesis, while *pboK* and *pboJ* only influence the amount of toxin produced. The three polycistronic units were transcribed at high levels at 18 °C but not at 28 °C, whereas gene *pboJ* was constitutively expressed. Together, our data suggest that the Pbo cluster synthesizes secondary metabolite(s), which could participate in the regulation of phaseolotoxin biosynthesis.

## 1. Introduction

*Pseudomonas syringae* pv. phaseolicola (syn. *P. amygdali* pv. phaseolicola), the causative agent of halo blight disease, is probably the most important bacterial pathogen of bean (*Phaseolus vulgaris*). The typical disease symptoms are small brown spots surrounded by a light-green or yellow halo [1]. This halo is produced by phaseolotoxin, a non-specific toxin that inhibits the ornithine carbamoyltransferase (OCTase) activity from plants, mammalian, and bacterial sources, causing a phenotypic requirement for arginine and polyamines [2,3]. This property led to the development of a rapid bioassay for evaluating the growth inhibition of an *Escherichia coli* indicator strain caused by the toxin contained in the pseudomonas bacterial culture [4]. Phaseolotoxin comprises two moieties: the inorganic moiety, N^δ^ N′-sulfodiaminophosphinyl, and the L-ornithyl-alanyl-homoarginine tripeptide [5,6]. Toxin production is regulated mainly by temperature, being optimally produced at 18 to 20 °C, while at 28 °C (the optimal growth temperature for *P. syringae*), it is not detected [7,8]. The phaseolotoxin is likely involved in the disease process because low temperatures inducing its biosynthesis are also conducive to disease and, additionally, because bean extracts stimulate toxin biosynthesis [1,9]. A chromosomal region of *P. syringae* pv. phaseolicola NPS3121, known as the Pht cluster, contains genes required for phaseolotoxin biosynthesis/regulation. This cluster comprises 23 genes organized in five transcriptional units, two monocistronic and three polycistronic [10]. However, the function of only a few of the *pht* genes has been experimentally demonstrated. Thus, genes *argK*, *amtA*, *phtQ* and *phtU* code for a resistant OCTase (ROCT), an L-arginine:lysine amidinotransferase, and two ATP grasp family peptide ligases, respectively [11,12,13,14,15].

Additionally, genes located outside the Pht cluster have been involved in phaseolotoxin regulation, such as the GacS/GacA two-component system [16,17]. The GacS/GacA system has proven necessary for fitness, lesion formation and symptom development by globally regulating pathogenicity and virulence factors. This regulatory system controls some toxins synthesized through pathways involving the activity of non-ribosomal peptide synthetases (NRPSs) or polyketide synthetases (PKSs) [18,19,20,21]. A putative NRPS, coded outside the Pht cluster by the gene *PSPPH_4550*, is controlled by GacA. The expression pattern of *PSPPH_4550* is the same as that observed for genes within the Pht cluster, induced at 18 °C. Additionally, this gene is necessary for phaseolotoxin production [16]. In *P. syringae*, NRPSs and PKSs are enzymes commonly involved in producing several toxins [22]. Archetypical NRPSs comprise an arrangement of modules, each composed of at least three different domains: the adenylation domain, which activates an amino acid, a peptidyl carrier protein domain that attaches the peptide and a condensation domain, which catalyzes the formation of the peptide bond between the activated amino acid and the growing peptide [23]. Coronatine, syringomycin, syringopeptin and mangotoxin are phytotoxins produced by different *P. syringae* pathovars whose biosynthesis involve NRPSs and PKSs [24].

Here, we determined that gene *PSPPH_4550* is included within a genomic island that is present in only a few other *P. syringae* strains, suggesting a possible horizontal transfer origin similar to that proposed for the Pht cluster [10,25,26,27,28]. The chromosomal fragment containing the gene *PSPPH_4550* from *P. syringae* pv. phaseolicola NPS3121 is part of a large cluster, the Pbo cluster, composed of 16 genes organized in four transcriptional units, three polycistronic and one monocistronic. As occurs with the Pht cluster, the *pbo* genes are thermoregulated and expressed at 18 °C but not at 28 °C, with the exception of the constitutively expressed gene *pboJ*. Mutation of nine of the *pbo* genes resulted in a Tox-minus phenotype for five of them, while four mutants exhibited low levels of toxin production. We proposed that the Pbo cluster is involved in the regulation of the biosynthesis of phaseolotoxin.

## 2. Results

### 2.1. Gene PSPPH_4550 Is Included within a Putative Genomic Island

**Figure 1:**
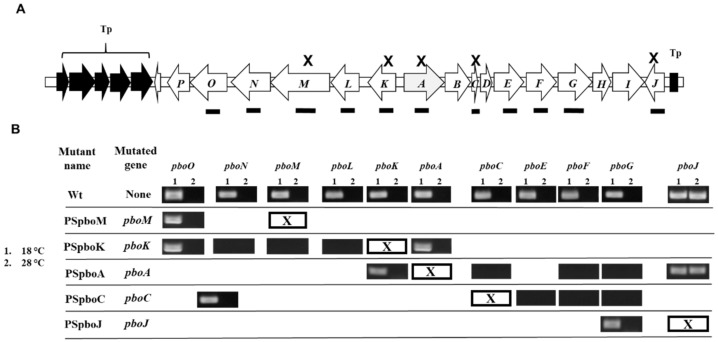
Pbo cluster of *P. syringae* pv. phaseolicola and expression patterns. (**A**) Schematic representation of the Pbo cluster. Genes are represented by block arrows, indicating the direction of transcription. Black bars represent the approximate size and position of amplicons obtained by RT-PCR to assess the transcription activity of different *pbo* genes. (**B**) Amplifications obtained by RT-PCR of diverse *pbo* genes are shown for the wild-type strain and *pbo* mutants at both 18 °C (1) and 28 °C (2); the mutated *pbo* gene is indicated by a white box with an X.

**Figure 2:**
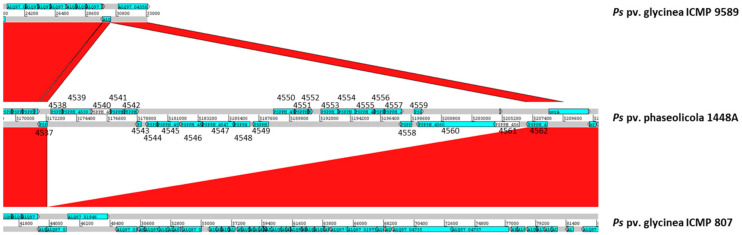
Gene *PSPPH_4550* is included in a putative genomic island. Graphical representation of a blastn comparison between contigs from *P. syringae* pv. glycinea ICMP 9589 (accession no. RBNP01000856) and *P. syringae* pv. glycinea ICMP 807 (accession no. RBNZ01000045), and the genome sequence of *P. syringae* pv. phaseolicola 1448A (accession no. CP000058), centered in the genomic region containing gene *PSPPH_4550*. Sequences were compared using blastn at the NCBI with default settings for megablast, and relevant areas were visualized using ACT with red shadings connecting direct homologous regions. Numbers indicate the corresponding PSPPH locus tag for relevant coding sequences of strain 1448A, which are indicated by blue arrows or, when they correspond to pseudogenes, by white arrows.

In *P. syringae* pv. phaseolicola 1448A, the putative NRPS gene *PSPPH_4550* is included in a large genomic region that contains several CDSs that show homology to, or contain, typical domains of transposases and recombinases (Figure 1A and Figure 2; Table 1). The software IslandViewer 4 predicts two overlapping putative genomic islands (positions 5,172,263..5,196,698 and 5,185,788..5,197,870 in accession no. CP000058) largely covering this genomic region. Moreover, a blastn comparison with draft genomes using the NCBI server indicates the presence of this genomic region in most strains of *P. syringae* pv. phaseolicola and in a few other strains of the *P. syringae* group. However, a continuous 34.8 kb fragment containing this region (positions 5,172,263..5,207,055) is missing, among others, in genome sequences from strains *P. syringae* pv. phaseolicola ICMP 5059 and 1664R, both isolated from *Vigna radiata*, and two strains of *P. syringae* pv. glycinea, although synteny to the genome of strain 1448A is otherwise largely maintained around the genomic island (Figure 2 and data not shown). Additionally, the genomic island is flanked by 18 nt imperfect direct repeats (positions 5,172,271..5,172,288 and 5,207,035..5,207,052); this is relevant because direct repeats are often associated to the ends of genomic islands and could be sites of recognition for mobility [29]. Together, these results, therefore, indicate that the gene *PSPPH_4550* is part of a large genomic island, which we named the Pbo genomic island.

**Figure 3:**
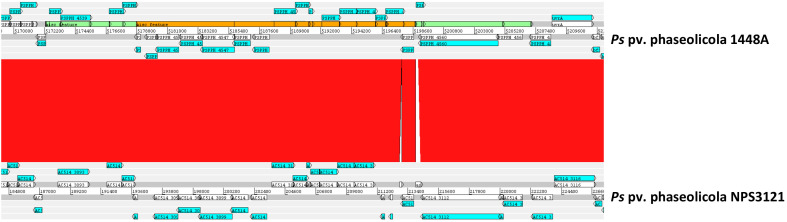
The genomic island containing the Pbo cluster is well conserved in *P. syringae* pv. phaseolicola strains 1448A and NPS3121. Graphical representation of a blastn comparison between the complete genome of *P. syringae* pv. phaseolicola 1448A (accession no. CP000058) and a contig from *P. syringae* pv. phaseolicola NPS3121 (accession no. LGKW01000003). Sequences were compared using blastn at the NCBI with default settings for megablast, and relevant areas were visualized using ACT, with red shadings connecting direct homologous regions. The extent of the Pbo genomic island and the Pbo cluster are indicated with green and orange boxes, respectively, in the genome of strain 1448A.

Within the genomic island, the gene *PSPPH_4550* is part of a cluster of 16 annotated CDSs surrounded by putative transposases (Figure 1A) (positions 5,178,760..5,198,744) and with an average 48% GC content, contrasting with the 57.8% GC for the 1448A chromosome. Modeling analyses with the Phyre^2^ server, together with domain analyses in the Pfam server, indicate that the deduced products of these 16 CDSs might participate in the biosynthesis of secondary metabolites (Table 1). Since *PSPPH_4550* is necessary for the biosynthesis of the phaseolotoxin [16], we undertook the genetic and functional characterization of this cluster, which we named here cluster Pbo (**P**haseolotoxin **b**iosynthesis **o**peron) and designated *PSPPH_4550* as *pboA*. The organization of the putative genomic island and the Pbo cluster is conserved with very high identity (≥99%) in strain NPS3121 (accession no. LGKW01000003), although its corresponding sequence contains two sequencing gaps flanking the last gene of the cluster (*pboJ*) (Figure 3).

### 2.2. Gene Expression Patterns

As it occurs with the *pht* genes, the expression of gene *pboA* occurs at 18 °C but is negligible at 28 °C [16]. We, therefore, evaluated by RT-PCR analyses if the other genes of the Pbo cluster also displayed a thermoregulated expression pattern. The results show that 10 of the genes analyzed, belonging to three of the four transcriptional units identified (see below), are transcribed at high levels at 18 °C but undetectable levels at 28 °C (Figure 1B). Only gene *pboJ*, which comprises the fourth transcriptional unit, also showed expression at 28 °C (Figure 1B).

### 2.3. The Pbo Cluster Is Organized in Four Transcriptional Units

**Figure 4:**
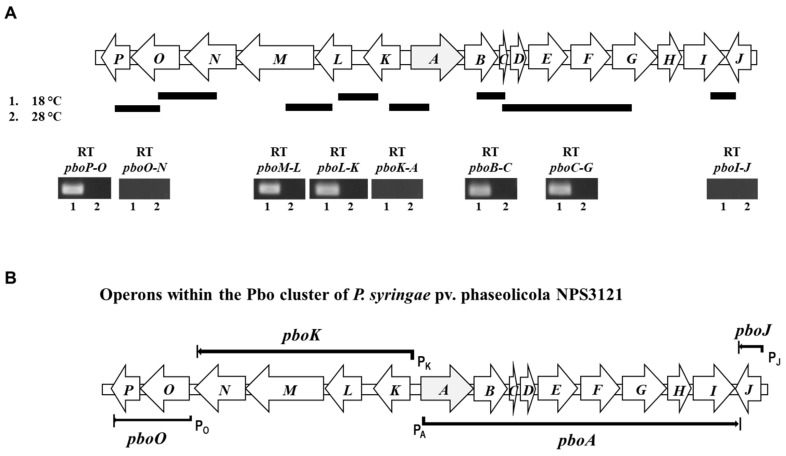
Reverse transcription-PCR analysis of the Pbo cluster and definition of transcriptional units. (**A**) Each RT-PCR amplification is shown and labeled with the amplicon name. The bars above the chromosomal fragment illustrate the amplicon. The numbers indicate the temperature at which bacteria were grown. (**B**) Proposed operon arrangement of the Pbo cluster of *P. syringae* pv. phaseolicola NPS3121. The Pbo cluster contains 16 genes organized into four transcriptional units, including one monocistronic and three polycistronic. The first polycistronic operon is from *pboA* to *pboI*; the second is from *pboK* to *pboN*; the third is from *pboO* to *pboP.* Operons are named after the first gene of the operon.

We first used RT-PCR analyses of contiguous genes to investigate the organization of *pbo* genes in transcriptional units (Figure 4A). Primers spanning *pboA–pboK* and *pboI*–*pboJ* did not produce any amplicon, whereas amplification from *pboB*–*pboC* and *pboC*–*pboG* was observed (Figure 4A). These results show that the genes *pboA* and *pboK* belong to different transcription units. Likewise, the genes *pboI* and *pboJ* are part of different operons. Concerning *pboN* and *pboO*, a primer pair overlapping both CDSs failed to produce amplification products, indicating that *pboN* and *pboO* are not co-transcribed. RT-PCR analyses also indicated that genes *pboO* and *pboP* are likely part of the same operon because they were transcribed together (Figure 4B), which was expected because they show overlapping translation stop and start codons.

To better define the operons, we evaluated the transcription pattern of *pbo* genes by RT-PCR in strains containing polar mutations inactivating genes *pboA*, *pboC*, *pboJ*, *pboK* and *pboM* (Figure 1B). The polar effect of the mutation on genes *pboA* and *pboC* affected the expression of genes located downstream up to at least gene *pboG*, which was the last gene examined by RT-PCR of this putative operon. Accordingly, genes *pboA* to *pboG* belong to the same operon. This operon also likely includes genes *pboHI* because the start codon of *pboH* is only four nucleotides downstream of the stop codon for *pboG*, whereas the start codon of *pboI* and the stop codon of *pboH* overlap.

As expected, because of its orientation, the transcription of *pboJ* was unaffected in strain PSpboA, containing a mutation in gene *pboA* (Figure 1B). Therefore, *pboJ* belongs to an independent transcriptional unit.

Similarly, a polar mutation on gene *pboK* prevented the generation of RT-PCR amplicons for the downstream genes up to *pboN*. However, the transcription of the gene *pboO* was not affected in polar mutants in genes *pboK* or *pboM* (Figure 1B). These results thus indicate that genes *pboK* to *pboN* belong to the same transcriptional unit. Additionally, the start codon for CDS *PSPPH_4543*, coding for a hypothetical protein, is situated 436 nt downstream of the stop codon of *pboP*, being, therefore, likely transcribed separately from *pboOP*.

All these results suggest that the genes coded into the Pbo cluster of *P. syringae* pv. phaseolicola NPS3121 are organized in at least four operons, including three polycistronic and one monocistronic. A large polycistronic operon encompasses nine genes, *pboA*, *pboB*, *pboC*, *pboD*, *pboE*, *pboF*, *pboG*, *pboH* and *pboI*. The second transcriptional unit contains *pboK*, *pboL*, *pboM* and *pboN* genes. The genes *pboO* and *pboP* are transcribed together, comprising another polycistronic operon. Finally, the monocistronic operon is comprised by gene *pboJ* (Figure 1B). All four identified transcriptional units are preceded by correctly spaced −35 and −10 boxes, as predicted by the software BPROM; however, using this software, we did not find any well-conserved transcription factor binding site receiving high scores in the promoter regions of the *pbo* operons (data not shown).

### 2.4. Production of Phaseolotoxin Requires the Participation of Pbo Genes

**Figure 5:**
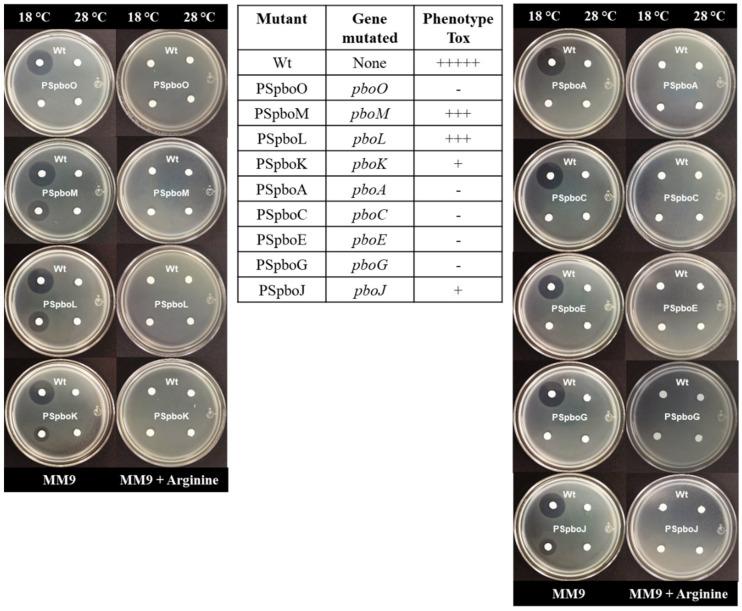
Production of phaseolotoxin by mutants of *P. syringae* pv. phaseolicola NPS3121 in diverse *pbo* genes. The table contains the mutated genes and the qualitative comparison between mutants and wild-type phaseolotoxin phenotype. Growth inhibition haloes that are reverted in media supplemented with arginine evidence production of phaseolotoxin. The photographs illustrate the growth inhibition assays. +++++, wild-type phaseolotoxin production; -, no phaseolotoxin produced; +, low phaseolotoxin level; +++, medium phaseolotoxin level.

We evaluated the involvement of the Pbo cluster in the biosynthesis of phaseolotoxin by an *E. coli* growth inhibition assay using strains with polar mutations in different genes of the cluster (see Materials and Methods). All mutants within the *pboA* transcriptional unit (strains PSpboA, PSpboC, PSpboE and PSpboG) as well as mutant PSpboO showed a Tox-minus phenotype (Figure 5). Conversely, mutants in the gene *pboJ* and within the *pboK* transcriptional unit (strains PSpboK, PSpboM and PSpboL) were still able to synthesize phaseolotoxin, although at a lower level than the wild-type strain and with less toxin production by mutants in genes *pboK* and *pboJ* (Figure 5).

### 2.5. The Pbo Cluster Has a Limited Distribution among Pseudomonads and Is Not Associated with the Phaseolotoxin Cluster

**Figure 6:**
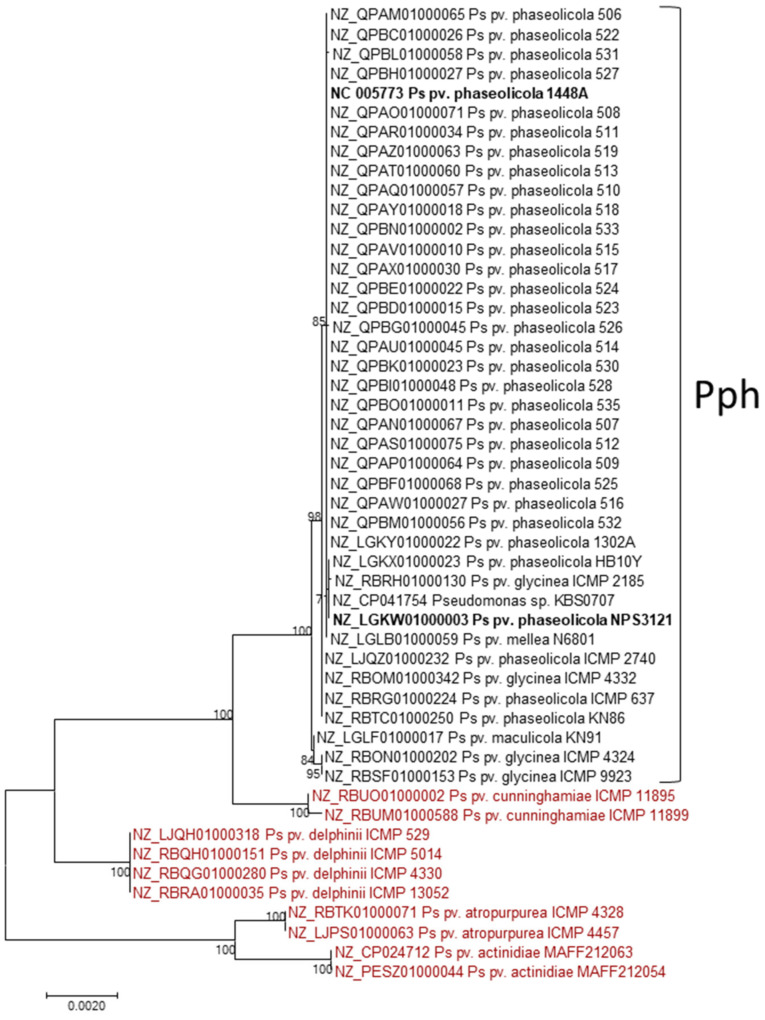
Conservation and phylogeny of the Pbo cluster among Gammaproteobacteria. The tree was constructed with contigs containing sequences with significant identity to at least 70% of the Pbo cluster from *P. syringae* pv. phaseolicola 1448A. Alignment of nucleotide sequences with Muscle (total of 14,121 nt), identification of the best model and construction of the maximum likelihood tree, using the Kimura 2-parameter model, were conducted with MEGA7. All positions containing gaps and missing data were eliminated. The tree is drawn to scale, with branch lengths measured in the number of substitutions per site. Numbers in branches indicate per cent bootstrap values with 100 replicates. The whole genome phylogeny of the strains included in the branch labelled as Pph, available at the NCBI, suggests that they all belong to *P. syringae* pv. phaseolicola and that some of them have been misclassified. Strains in black contain both the Pbo and the Pht (phaseolotoxin biosynthesis) clusters, whereas those strains in red only contain the Pbo cluster.

The nucleotide sequence of the Pbo cluster (19,985 nt) was used as query in a discontiguous megablast search against the RefSeq Genome Database of Gammaproteobacteria at the NCBI server. This search returned only 50 genomes with a continuous query coverage over 70%, plus a few other genomes with smaller coverages over several small contigs, all belonging to *P. syringae sensu lato* (Figure 6 and data not shown). Nearly all of the *P. syringae* pv. phaseolicola genomes in the database, all of which contained the Pht cluster, displayed over 99.9% identity with 100% query coverage. Exceptions were strains ICMP 5059 and 1644R, not containing the Pbo cluster, and strains Y5_2 and K4, which contained the Pbo cluster with diverse internal deletions. We do not know if these four strains are able to synthesize phaseolotoxin.

Strains of *P. syringae* pv. actinidiae biovars 1 and 6 synthesize phaseolotoxin and contain a biosynthesis cluster that is nearly identical to that of *P. syringae* pv. phaseolicola strains [30,31]. However, we did not find the complete Pbo cluster in any of the sequenced genomes from these biovars, and only six strains from biovar 6 contained sequences covering less than 45% of the Pbo cluster. Additionally, the genomes of 10 other strains from different *P. syringae* pathovars not containing the phaseolotoxin cluster also contained sequences displaying over 98% identity with over 97% query coverage, spanning one to three contigs, to the Pbo cluster.

These results indicate that the Pbo cluster has been recently transferred horizontally among strains of *P. syringae*, having a limited distribution within this species complex, and that its possession does not correlate with the possession of the cluster for the biosynthesis of phaseolotoxin. Therefore, the Pbo cluster likely participates in other cellular processes in addition to the biosynthesis of phaseolotoxin.

## 3. Discussion

We identified a 34.8 kb genomic island in *P. syringae* pv. phaseolicola NPS3121 containing the newly described Pbo cluster. This cluster spans approximately 20 kb, contains 16 genes organized in four transcriptional units and is involved in the biosynthesis of the antimetabolite toxin phaseolotoxin.

The Pbo cluster includes the gene *pboA* (locus_tag *PSPPH_4550*), which codes for a putative non-ribosomal peptide synthetase and was previously shown to be expressed at 18 °C, but not at 28 °C; additionally, a polar mutation in *pboA* abolished the production of phaseolotoxin [16]. Extending these results, we show here that the three polycistronic transcriptional units of the Pbo cluster are thermoregulated (Figure 3). Likewise, they are also involved in the biosynthesis of phaseolotoxin, although with a differential contribution (Figure 2): operons *pboA* and *pboO* are both essential for toxin biosynthesis, whereas mutations in operon *pboK* only cause a reduction in the amount of phaseolotoxin produced. We do not yet have any satisfactory explanation for this differential requirement of Pbo genes for the biosynthesis of phaseolotoxin. The gene *pboJ* is transcribed as a monocistronic unit and mutations in this gene cause a reduction in the amount of phaseolotoxin produced. Unlike other genes from the Pbo cluster, however, this gene is expressed constitutively. These differential patterns of expression and involvement in the production of the toxin of genes within the Pbo cluster are not surprising. In fact, the Pht cluster contains the genes directly involved in the thermoregulated biosynthesis of phaseolotoxin; however, not all the genes within the cluster are essential for the synthesis of the toxin and not all of them show a thermoregulated expression pattern [10]. In summary, both the Pht and the Pbo clusters are thermoregulated and essential for the biosynthesis of phaseolotoxin. Additionally, the Pht cluster and, at least, operons *pboA* (genes *pboA* and *pboN*) and *pboK* (genes *pboEFG*) from the Pbo cluster are regulated by the GacS/GacA two-component system [16].

Based on the conserved domains and annotation of the individual genes, the Pbo cluster is likely involved in the biosynthesis of a secondary metabolite(s) resulting from the action of NRPSs and PKSs. Specialized NRPSs synthesize non-ribosomal peptides and commonly contain several elongation modules, which typically consists of three domains: the adenylation (A) domain, the peptidyl carrier protein (PCP) or thiolation domain and the condensation (C) domain, all of which are vital for the production and bioactivity of NRPSs [32]. In some cases, terminal modules are also included, such as reductase (R) or thioesterase (Te) domains, which release the final peptide [33]. According to our Phyre^2^ and Pfam analyses, the deduced products of several of the *pbo* genes contain conserved domains typical of NRPSs (Table 1). For example, PboA contain A and C domains, PboC a PCP domain and PboO a C domain. Additionally, PboB is similar to a beta-ketosynthase from the R1128 polyketide biosynthetic pathway, whereas the product of the gene *pboM* showed a domain similar to a ketosynthase-acyltransferase di-domain of polyketide synthase. The polyketides are a large class of secondary metabolites produced by multifunctional PKSs, which are generally coded for by genes organized in gene clusters [34].

Our data strongly support the hypothesis that the Pbo cluster is included in a *bona fide* genomic island and has been acquired during a horizontal gene transfer event [16,27]. First, the genomic island is flanked by genes coding transposases and direct repeats; second, the GC content of the Pbo cluster (48%) is considerably lower that the GC content of the chromosome of *P. syringae* pv. phaseolicola 1448A (57.8%); third, a blast comparison indicates that the genomic island is present in only a few strains of *P. syringae sensu lato* (Figure 2). The Pbo island is bordered by a Tn*7*-like transposon and a resolvase (Table 1), and thus has a different structure than the genomic island containing the Pht cluster [28]. Remarkably, there is no correlation between the possession of the Pbo genomic island and the phaseolotoxin biosynthesis cluster; thus, there are strains that contain both, only one or neither (Figure 6 and data not shown). In turn, this implies that there are *P. syringae* strains that do not require the Pbo cluster for the biosynthesis of phaseolotoxin. In particular, previous results [16] and our own analyses (Figure 6) demonstrated the absence of the Pbo cluster in strains of *P. syringae* pv. actinidiae that produce phaseolotoxin [30,31].

We predict two possible functions for the Pbo cluster. An obvious possibility is that the cluster is involved in the biosynthesis of the organic moiety of the toxin, which could require the activity of NRPSs for the assembly of the tripeptide. A relevant weakness of this hypothesis is that there are strains that are capable of synthesizing phaseolotoxin but do not contain the Pbo cluster, such as strains of *P. syringae* pv. actinidiae. However, we could then argue that different strains could produce phaseolotoxin with different chemical structures. In fact, an analogue of phaseolotoxin [(N^δ^-phosphosulphamyl)-ornithylserylhomoarginine] is produced as a minor component (5–10% of the total toxin) by phaseolotoxin-producing strains of *P. syringae* pv. phaseolicola [7,35]. Nevertheless, this is a minor structural modification that substitutes alanine by serine, only differing in that one of the methylenic hydrogens is replaced in serine by a hydroxyl group. Additionally, the structure of phaseolotoxin from a strain of pathovar actinidiae coincides with that of purified phaseolotoxin from *P. syringae* pv. phaseolicola [36]. Therefore, the conservation of a main toxin structure among phaseolotoxin producers seems the most plausible scenario; thus, it seems unlikely that the Pbo cluster would directly participate in the biosynthesis of the phaseolotoxin molecule.

A second more realistic possibility is that the Pbo cluster is participating in the regulation of phaseolotoxin biosynthesis, particularly considering the complexity of the regulatory circuitry involved. This putative regulatory activity could then be similar to the regulation of the antimetabolite toxin mangotoxin by the volatile compound leudiazen, which belongs to the family of diazeniumdiolate communication molecules [37]. The biosynthesis of leudiazen requires the *mgoBCAD* operon, which consists of four genes coding for two predicted oxygenases, an NRPS and a putative polyketide cyclase/dehydratase [38]. Besides mangotoxin biosynthesis, leudiazen also impacts virulence and likely other phenotypic traits, and this is likely the reason why homologs of the *mgo* operon are present in diverse pseudomonads and other bacteria [37,39]. Thus, it is conceivable that the putative secondary metabolite produced by the Pbo cluster is also a signaling molecule regulating phaseolotoxin biosynthesis; likewise, its potential implication in regulating other phenotypes would also explain the presence of the Pbo island in bacteria that do not synthesize phaseolotoxin.

## 4. Materials and Methods

### 4.1. Bacterial Strains, Media and Growth Conditions

The bacterial strains used in this study are listed in Table 2. *P. syringae* pv. phaseolicola NPS3121 wild-type strain and mutant derivatives were grown at 18 or 28 °C on King’s B medium (KB) [40] or modified M9 minimal medium (MM9) [41], containing (final concentration) 0.4 mM CaCl_2_, 4 mM MgSO_4_ and 8 g L^−1^ of sucrose as a carbon source. *Escherichia coli* strain DH5α was grown in Luria-Bertani medium at 37 °C. When required, the following antibiotics were added (final concentrations in µg mL^−1^): kanamycin (Km), 100, carbenicillin (Cb), 300, and rifampicin (Rif), 100.

### 4.2. Molecular Biology Techniques and Bioinformatics

Molecular biology techniques such as purification of plasmid DNA, chromosomal DNA extraction, agarose gel electrophoresis, transformation, DNA restriction and polymerase chain reaction (PCR) were performed as previously described [42]. Chromosomal DNA from *P. syringae* pv. phaseolicola was purified using a simple method [45]. Plasmids and restriction enzymes were used according to instructions provided by the suppliers (Invitrogen, Waltham, MA, USA). The genome sequence of *P. syringae* pv. phaseolicola 1448A was obtained from the NCBI GenBank (accession no. CP000058.1) and was used as reference [46]. The oligonucleotides were designed using Vector NTI software (Invitrogen, Waltham, MA, USA).

The Phyre^2^ server (Protein Homology/analogy Recognition Engine V 2.0) was used to predict and analyze the structure and function of putative protein products [47]. Prediction of conserved protein domains was also examined using the Pfam server at the EMBL-EBI [48]. The MEGA7 software (v. 7.0.26) [49] was used for phylogenetic reconstructions, including multiple-sequence alignments with the MUSCLE program, determining the optimal substitution model, and construction of maximum-likelihood phylogenetic trees; confidence levels of the branching points were determined using 100 bootstraps replicates. Promoters were predicted using the online BPROM server (http://www.softberry.com; accessed on February 2021).

### 4.3. Construction of P. syringae pv. Phaseolicola NPS3121 Mutants

*P. syringae* pv. phaseolicola mutants were obtained using a previously described gene inactivation method with slight modifications [50] and using specific oligonucleotides (Table 3). DNA fragments corresponding to internal coding sequences (CDSs), thus lacking the 5′ and 3′ ends, were amplified by PCR using purified chromosomal DNA from *P. syringae* pv. phaseolicola NPS3121 as template and cloned into the commercial suicide vector pCR4-TOPO (Invitrogen, Waltham, MA, USA). The resulting constructions were electroporated into *P. syringae* pv. phaseolicola NPS3121 for gene disruption by integration; this procedure resulted in mutants with polar effect on downstream genes of the same operon. These mutations of the *pbo* genes were verified by PCR and RT-PCR analyses to corroborate gene disruption and expression. We could not detect gene expression of any of the mutated genes in their mutant background (data not shown). Thus, mutants with polar effect were obtained inactivating genes *pboA*, *pboC*, *pboE*, *pboG*, *pboJ*, *pboK*, *pboL*, *pboM* and *pboO* (Table 2).

### 4.4. Phaseolotoxin Bioassays

Phaseolotoxin production by *P. syringae* pv. phaseolicola wild-type and mutant strains was evaluated by the *E. coli* growth inhibition assay using *E. coli* JM103 as indicator, essentially as described [10,51]. *P. syringae* pv. phaseolicola strains were inoculated in MM9 until an initial OD_600_ of 0.01 and incubated at 18 °C or 28 °C for 72 h. The supernatants were then recovered by centrifugation at 5000× *g* for 10 min, and aliquots deposited on 6 mm Whatman antibiotic assay discs over double layer MM9 plates seeded with the indicator strain. Production of phaseolotoxin was confirmed by reversion of inhibition haloes in MM9 plates supplemented with 10 mM arginine.

### 4.5. RNA Extraction and Reverse Transcription-PCR Analysis

RNA was purified from strains of *P. syringae* pv. phaseolicola grown in MM9 at 18 or 28 °C. Total RNA was purified using TRIzol reagent as suggested by the supplier (Invitrogen, Waltham, MA, USA). RNase-free DNase (Invitrogen, Waltham, MA, USA) was used to remove the genomic DNA. The RNA was checked for integrity in denaturing agarose gel and used for reverse transcription (RT) and PCR (RT-PCR) using the SuperScript™ III One-Step RT-PCR System with Platinum™ *Taq* DNA Polymerase (Invitrogen, Waltham, MA, USA). Controls were: (a) PCR without the reverse transcription step, to verify the absence of DNA; (b) RT-PCRs without RNA templates, to detect any contaminating DNA/RNA; (c) PCRs using chromosomal DNA as a template, to ensure primer fidelity; (d) the amplification of a portion of the 16S rDNA operon using suitable primers, as an internal control of the reaction. The RT reactions were conducted at 55 °C for 30 min, followed by PCR amplification at 94 °C for 2 min for 1 cycle; 94 °C for 15 s, 60 °C for 30 s and 68 °C for 2 min for 30 cycles; 68 °C for 5 min for 1 cycle.

## Figures and Tables

**Table 1:** Conserved domains found during Phyre^2^ and Pfam analyses of predicted proteins encoded by the Pbo cluster and the flanking coding sequences.

Gene	Locus_Tag	Product	Conserved Domains (Confidence)	Annotation or Pfam Domain(s), Pfam Clan (E-Value) ^a^
	*PSPPH_4538*	AAZ34646.1	Catalytic component of the Tn7 transposition system (100)	Transposon Tn7-like transposase protein A, CL0236 (2.2 × 10^−22^)
	*PSPPH_4539*	AAZ33379.1	Bacteriophage Mu transposase core domain (100)	Transposon Tn7-like transposase protein B, Integrase core domain, CL0219 (1.2 × 10^−3^)
	*PSPPH_4540*		Transposon Tn7-like transposition protein C (100)	Pseudogene
	*PSPPH_4541*	AAZ34404.1	Transposase TniQ. Transposition of the mercury-resistance (99.9)	TniQ (8.4 × 10^−7^)
	*PSPPH_4542*	AAZ37460.1	SAP domain (51.8)	Tn7-like transposition protein D (4.9 × 10^−4^)
	*PSPPH_4543*	AAZ36781.1	RecO N-terminal domain-like (25.4)	Hypothetical protein
*pboP*	*PSPPH_4544*	AAZ33084.1	Epoxide hydrolase (100)	Alpha/beta hydrolase family, CL0028 (3.3 × 10^−10^)
*pboO*	*PSPPH_4545*	AAZ34142.1	Synthetase c, a nonribosomal peptide synthetase termination module (100)	Condensation domain, CL0149 (2.7 × 10^−3^)
*pboN*	*PSPPH_4546*	AAZ33594.1	Initiation module of LgrA in the thiolation2 state (100)	AMP-binding enzyme, CL0378 (8.5 × 10^−80^), CL0531 (2.3 × 10^−6^)
*pboM*	*PSPPH_4547*	AAZ34320.1	Ketosynthase- acyltransferase di-domain from module CurL of the curacin, a polyketide synthase (100)	Beta-ketoacyl synthase, CL0046 (3.7 × 10^−56^)
*pboL*	*PSPPH_4548*	AAZ35215.1	Oxidoreductase from *Streptomyces* sp. in complex with FADH_2_ and glycerol (100)	Acyl-CoA dehydrogenase, CL0087 (6.1 × 10^−18^)
*pboK*	*PSPPH_4549*	AAZ32977.1	Glutamine synthetase from *Bacillus subtilis* (44.5)	Hypothetical protein
*pboA*	*PSPPH_4550*	AAZ34936.1	Condensation and adenylation domains of teixobactin-2 producing nonribosomal peptide synthetase Txo2 serine module (100)	AMP-binding enzyme, CL0378 (7.2 × 10^−67^), CL0531 (6.9 × 10^−10^)
*pboB*	*PSPPH_4551*	AAZ34497.1	Priming beta- ketosynthase from the R1128 polyketide biosynthetic pathway (100)	3-Oxoacyl-[acyl-carrier-protein (ACP)] synthase III, CL0046 (1.3 × 10^−7^)
*pboC*	*PSPPH_4552*	AAZ37563.1	Thiolation-reductase di-domain from an archaeal non-2 ribosomal peptide synthetase (99.8)	Phosphopantetheine attachment site, CL0314 (1.3 × 10^−8^)
*pboD*	not annotated	WP_057456395.1	Uncharacterized protein ECA2234 (53.9)	Hypothetical protein
*pboE*	*PSPPH_4553*	AAZ32971.1	Structure of *E. coli* YajR transporter (100)	Major Facilitator Superfamily, CL0015 (9.4 × 10^−29^)
*pboF*	*PSPPH_4554*	AAZ35479.1	Lysine-2,3 aminomutase from *Clostridium* *subterminale* Sb4 (100)	Radical SAM protein, 4Fe-4S single cluster domain, CL0036 (4.1 × 10^−11^), CL0344 (7.9 × 10^−5^)
*pboG*	*PSPPH_4555*	AAZ36161.1	Structure of L-amino acid ligase from *Bacillus* *licheniformis* (100)	ATP-grasp domain, CL0179 (2.9 × 10^−10^)
*pboH*	*PSPPH_4556*	AAZ34118.1	Isoleucine-4-hydroxylase. Structure of Ido from *Bacillus thuringiensis* (100)	2OG-Fe dioxygenase, CL0029 (7.8 × 10^−34^)
*pboI*	*PSPPH_4557*	AAZ35362.1	*E. coli* D-galactonate:proton symporter mutant E13 (100)	Major Facilitator Superfamily, CL0015 (1.0 × 10^−13^)
*pboJ*	*PSPPH_4558*	AAZ34790.1	NADH pyrophosphatase from *E. coli* K12 (99.9)	NUDIX domain, NUDIX hydrolase domain, CL0261 (3.2 × 10^−17^)
	*PSPPH_4559*	AAZ36496.1	Bipartite DNA-binding domain of Tc32 transposase bound to transposon DNA (99.2)	Helix-turn-helix domain of resolvase, CL0123 (7.4 × 10^−6^)

^a^ Independent E-value.

**Table 2:** Bacterial strains and plasmids.

Strain or Plasmid	Relevant Characteristics	Reference or Source
Bacterial strains		
*Escherichia coli*		
DH5α	F^–^*endA1 glnV44 thi-1 recA1 relA1 gyrA96 deoR nupG purB20* [φ80*lacZ*ΔM15] Δ(*lacZYA-argF*)*U169 hsdR17(r_K_^−^m_K_^+^*) λ*^−^*	[42]
JM103	*endA1 glnV44 sbcBC rpsL thi-1* Δ(*lac-proAB*) F′[*traD36 proAB^+^ lacI^q^ lacZ*ΔM15]	[43]
*P. syringae* pv. phaseolicola		
NPS3121	Rif^r^; wild-type strain, Tox^+^	[44]
PSpboO	Km^r^ Cb^r^; *pboO* mutant of NPS3121	This study
PSpboM	Km^r^ Cb^r^; *pboM* mutant of NPS3121	This study
PSpboL	Km^r^ Cb^r^; *pboL* mutant of NPS3121	This study
PSpboK	Km^r^ Cb^r^; *pboK* mutant of NPS3121	This study
PSpboA	Km^r^ Cb^r^; *pboA* mutant of NPS3121	This study
PSpboC	Km^r^ Cb^r^; *pboC* mutant of NPS3121	This study
PSpboE	Km^r^ Cb^r^; *pboE* mutant of NPS3121	This study
PSpboG	Km^r^ Cb^r^; *pboG* mutant of NPS3121	This study
PSpboJ	Km^r^ Cb^r^; *pboJ* mutant of NPS3121	This study
Plasmid pCR^®^4-TOPO^®^	Km^r^ Cb^r^; 3.95 kb	Invitrogen

Rif^r^, rifampicin resistance; Km^r^, kanamycin resistance; Cb^r^, carbenicillin resistance.

**Table 3:** Primers used in this study.

Gene	Primer Name	Primer Sequence (5′ → 3′)
*pboO*	S126d	GCCGTTGTGATAGCCGACAGTGA
	S127c	AACGCCAGCGCTTCATCCTTGT
*pboM*	S155d	GCTGCCTACGGCACAGGCATTGG
	S156c	GCGATTATGCCATCGTTGCTGCG
*pboL*	S136d	CCACGCTGGACAACATGGTGATC
	S137c	CATACTTTCTGGCCGCTACCCATTC
*pboK*	S122d	CATCTGTTCCAGCCGACGCAGA
	S123c	AACCCCGCGATCCTACAGACAGC
*pboA*	S134d	GCAAATTGCCAGTTGCGTTGCC
	S135c	CCTTTCGGTGTACCGGTAGAACCAG
*pboC*	S130d	GGGGTCATGCACCCGACACTTG
	S131c	CGTGCTTATCTGTGCCGATCGAGT
*pboE*	S157d	GCAGGCGCTGGTGATTGGCTTG
	S158c	GACACCAGCATGACCGGGATCTCG
*pboG*	S159d	CAGCGAGCCGGTCACTGAGCATC
	S160c	CCTGATTGAGCGCAATGCCGC
*pboJ*	S132d	TCTGTTCTGCAGCCTCAACGTGG
	S133c	TGAGCTGGACAAATTCAATGGAGTGA

## Data Availability

The data presented in this study are available upon request to the corresponding author.
